# The impact of social engagement on health-related quality of life and depressive symptoms in old age - evidence from a multicenter prospective cohort study in Germany

**DOI:** 10.1186/s12955-017-0715-8

**Published:** 2017-07-14

**Authors:** André Hajek, Christian Brettschneider, Tina Mallon, Annette Ernst, Silke Mamone, Birgitt Wiese, Siegfried Weyerer, Jochen Werle, Michael Pentzek, Angela Fuchs, Janine Stein, Tobias Luck, Horst Bickel, Dagmar Weeg, Michael Wagner, Kathrin Heser, Wolfgang Maier, Martin Scherer, Steffi G. Riedel-Heller, Hans-Helmut König, for the AgeCoDe Study Group, Wolfgang Maier, Martin Scherer, Heinz-Harald Abholz, Cadja Bachmann, Horst Bickel, Wolfgang Blank, Hendrik van den Bussche, Sandra Eifflaender-Gorfer, Marion Eisele, Annette Ernst, Angela Fuchs, André Hajek, Kathrin Heser, Frank Jessen, Hanna Kaduszkiewicz, Teresa Kaufeler, Mirjam Köhler, Hans-Helmut König, Alexander Koppara, Carolin Lange, Hanna Leicht, Tobias Luck, Melanie Luppa, Manfred Mayer, Edelgard Mösch, Julia Olbrich, Michael Pentzek, Jana Prokein, Anna Schumacher, Steffi Riedel-Heller, Janine Stein, Susanne Steinmann, Franziska Tebarth, Michael Wagner, Klaus Weckbecker, Dagmar Weeg, Jochen Werle, Siegfried Weyerer, Birgitt Wiese, Steffen Wolfsgruber, Thomas Zimmermann

**Affiliations:** 1 Department of Health Economics and Health Services Research, Hamburg Center for Health Economics, University Medical Center Hamburg-Eppendorf, Hamburg, Germany; 2 Department of Primary Medical Care, Center for Psychosocial Medicine, University Medical Center Hamburg-Eppendorf, Hamburg, Germany; 3 Institute of General Practice, Hannover Medical School, Hannover, Germany; 4 Central Institute of Mental Health, Medical Faculty Mannheim/Heidelberg University, Mannheim, Germany; 5 Institute of General Practice, Medical Faculty, Heinrich-Heine-University Düsseldorf, Düsseldorf, Germany; 6 Institute of Social Medicine, Occupational Health and Public Health, University of Leipzig, Leipzig, Germany; 7 Department of Psychiatry, Technical University of Munich, Munich, Germany; 8 Department of Psychiatry, University of Bonn, Bonn, Germany; 9 German Center for Neurodegenerative Diseases (DZNE), Bonn, Germany

**Keywords:** Depressive symptoms, EQ-VAS, Geriatric Depression Scale, Health-related quality of life, Old age

## Abstract

**Background:**

Thus far, only a few longitudinal studies investigated the impact of social engagement on health-related quality of life (HRQoL) and depressive symptoms in old age. Therefore, we aimed to examine the impact of social engagement on HRQoL and depressive symptoms in late life.

**Methods:**

Individuals aged 75 years and over at baseline were interviewed every 1.5 years in a multicenter prospective cohort study in Germany. While HRQoL was quantified by using the Visual Analogue Scale (EQ VAS) of the EQ-5D instrument, depressive symptoms was assessed by using the Geriatric Depression Scale (GDS). Individuals reported the frequency (“never” to “every day”) of social engagement (e.g., engagement in the church, as a volunteer, in a party, or in a club) in the last four weeks. Fixed effects regressions were used to estimate the effect of social engagement on the outcome variables.

**Results:**

After adjusting for age, marital status, functional status and chronic diseases, fixed effects regressions revealed that the onset of social engagement markedly increased HRQoL and considerably decreased depressive symptoms in the total sample and in women, but not men.

**Conclusions:**

Our findings corroborate the relevance of social engagement for HRQoL and depressive symptoms in old age. Encouraging the individuals to start, maintain and expand social engagement in late life might help to maintain and improve HRQoL and decrease depressive symptoms.

## Background

Social aspects of life can be divided into three components: support, networks and engagement [1]. While social support can be defined as the access to relationships that meet a person’s basic social needs through environmental supplies of social support [2], the use of social network can be defined as “an analytic concept, used to describe the structure of linkages between individuals or groups of individuals. Such networks have a variety of functions of which the provision of social support is but one” [3].

Social engagement, reflects an active engagement implying that individuals choose to participate [4, 5]. In 2009, 20% of the individuals aged 75 and above were socially engaged in Germany. These individuals were often engaged in activities connected to a church or a religion (33%), in the social (e.g. charity or another aid agency)/health sector (e.g. assisting with the nursing of sick people) (34%) or in other activities (e.g. sport club, theatre group, club for older people) (57%). Moreover, 20% of these socially engaged individuals were willing to expand their engagement. Furthermore, 18% of the individuals who were not socially engaged in 2009 were willing to start voluntary activities (4% = “yes”; 14% = “maybe”). Additionally, it was extremely important for individuals in late life to help other people in their voluntary activities [6]. Due to these reasons and as it is expected that the number and proportion of individuals in old age will considerably increase in the next decades, there is a huge potential for older people getting socially involved, e.g. in the issue of refugees. Particularly this issue is projected to be a challenge to Germany in upcoming decades, underlining the importance of social engagement in old age in the future.

Social engagement is also of particular relevance in old age since many events occur in old age such as retirement, the empty nest phenomenon or the death of the spouse or close friends. Moreover, it is worth emphasizing that the absence of social engagement is associated with main adverse health outcomes such as cognitive decline [7] or mortality [1].

However, the impact social engagement has on other main health outcomes including health-related quality of life (HRQoL) and depressive symptoms longitudinally is largely unknown. While HRQoL is a multidimensional construct, focusing on how an illness and its treatment affect physical, psychological, and social aspects of life, depressive symptoms include our psychological, emotional and social well-being [8].

Several cross-sectional studies found positive associations between social engagement and HRQoL as well as less depressive symptoms in old age across cultures [9, 10]. However, these studies fail to identify causal mechanisms. While some longitudinal studies exist investigating the impact of social variables on HRQoL and depressive symptoms in old age [11–13], most of them did not use panel data methods. Consequently, the causal association between social engagement and HRQoL as well as depressive symptoms is not well understood.

Moreover, most of the existing longitudinal studies focus on social support and social networks, including [14] informal caregiving [15], whereas only a few studies [12, 13, 16, 17] investigated the association between engagement and HRQoL as well as depressive symptoms. For example, Schwingel et al. [13] found that at baseline and 2-year follow up volunteering retirees had less depressive symptoms than non-volunteering retirees. Moreover, Hong et al. [16] found that volunteering was associated with longitudinal changes in depression (single item). Longitudinal studies investigating the impact of social engagement on HRQoL and depressive symptoms in late life are missing for Germany.

Therefore, to close this research gap, we aimed at investigating the impact of social engagement on HRQoL and depressive symptoms in late life. It is assumed that social engagement might provide a sense of competence and feelings of usefulness [18]. Moreover, these factors are associated with sense of control and mastery [19]. These characteristics are in turn related to HRQoL and depressive symptoms [20].

To address this research question adequately, data were derived from a multicenter prospective cohort study in Germany. Moreover, fixed effects (FE) regressions were used (i) to produce consistent estimates (time-constant unobserved factors were taken into account) and (ii) to gain insights into the causal relationship between social engagement and the outcome variables. More specifically, unlike other widely used panel data methods, FE regressions provide consistent estimates even if time-constant unobserved factors (such as genetic disposition) are correlated with the predictors - which is often the case in HRQoL and depressive symptoms research.

This knowledge about the longitudinal predictors is vital as the results of our study might highlight the importance of developing strategies to encourage social engagement in late life. This might help to preserve and increase HRQoL and decrease depressive symptoms in old age.

## Methods

### Sample

Data from the German Study on Ageing, Cognition and Dementia in Primary Care Patients (AgeCoDe) were used. The AgeCoDe study is a multicenter (Leipzig, Hamburg, Dusseldorf, Mannheim, Bonn and Munich) prospective cohort study. In 2003/2004 (baseline) individuals were recruited via General Practitioners’ (GP) offices. From that point on, individuals were interviewed by trained staff every 1.5 years (follow-up (FU) wave 5 taking place in 2011/2012). Inclusion criteria at baseline were: (i) ≥ 75 years, (ii) absence of dementia and (iii) at least one contact with the GP in the last 12 months. Exclusion criteria at baseline were: insufficient knowledge of the German language, consultations only via home visits, residence in a nursing home (NH), severe illness the GP would deem fatal within 3 months, deafness, blindness, lack of ability to provide informed consent and being an irregular patient of the participating practice. Luck et al. [21] published more details concerning the sampling frame. The study has been approved by the local ethics boards of all participating centers and written informed consent was obtained from all patients.

In our main analysis, four waves (FU wave 2 to FU wave 5, with n_FU Wave2_ = 2364) were used because HRQoL and comorbidity was included from FU wave 2 onwards. Main reasons for lack of follow-up data were refused participation and death.

### Health-related quality of life

By using the Visual Analogue Scale (EQ VAS) of the EQ-5D instrument [22], *HRQoL* was assessed. The scale ranges from 0 (worst imaginable health state) to 100 (best imaginable health state). This scale is frequently used to quantify self-reported health and has good psychometric properties in late life [23].

### Depressive symptoms

The Geriatric Depression Scale (GDS [24]) was used to quantify *depressive symptoms*, ranging from 0 (no depressive symptoms) to 15 (severe depression). The GDS also has good psychometric properties [25].

### Independent variables

Individuals reported the frequency (“every day”, “several times a week”, “once a week” and “never”) for *social engagement* in the last four weeks (e.g. tutoring, engagement in the church, as a volunteer, in a party, in an old-age home, or in a club). As for sociodemographic variables, *age*, *sex*, *education* (CASMIN classification [26]), with primary, secondary and tertiary education), *living situation* (Ref.: living alone in private household; others (with spouse/partner, with other relatives, nursing home, assisted living, retirement home, other)) and *marital status* (Ref.: married; others (single, widowed, divorced)) were used. Education and living situation were solely used for descriptive purposes.

*Functional impairment* was quantified by the Lawton and Brody Instrumental Activities of Daily Living scale (IADL, 0 = worst score, 8 = best score) [27]. The *comorbidity* was quantified by the presence of 28 chronic conditions (assessed by the GP) such as diabetes, or obesity. If a chronic condition was present, the GP rated the severity from 1 (= mild) to 4 (= severe). By summing the severity rating for chronic conditions as present, a weighted count comorbidity score was computed to take the severity of the chronic diseases into consideration.

### Statistical analysis

In order to estimate the effects of time-dependent regressors on our outcome variables (HRQoL and depressive symptoms), linear fixed effects (FE) regressions were used. Contrarily to FE regressions, random effects (RE) regressions are inconsistent if time-constant unobserved variables such as optimism, personality or genetic disposition are correlated with the regressors [28]. Sargan-Hansen tests (which is a Hausman test with cluster-robust standard errors) indicated that FE regressions are the method of choice. This means that FE regressions provide consistent estimates even if time-constant factors are correlated with the predictors (under the assumption of strict exogeneity). The FE-estimator is also called ‘within-estimator’ as solely intraindividual changes over time are exploited. Furthermore, it is worth noting that gender-specific regressions were computed since social engagement varies between women and men [29, 30]. The level of significance was set to 5%. Statistical analyses were performed using Stata 14 (Stata Corp., College Station, Texas). Robust standard errors that cluster errors at the individual level were computed. It is worth noting that the Stata command for FE regression analysis include subjects with only one observation in calculating the number of observations since these subjects provide information about, e.g., the variance components, the constant, the between R^2^, and the overall R^2^. Nevertheless, it does not affect the beta-coefficients as well as the standard errors.

## Results

### Descriptive analysis

At FU wave 2, mean age was 82.5 years (±3.4 years) (see Table 1). The majority was female (65.8%), had a low education (60.5%), were not married (61.4%) and was living alone in private household (51.4%). The mean comorbidity (weighted count score) was 4.5 (±3.8), IADL was 6.8 (±1.7), mean EQ-VAS was 65.5 (±17.9) and mean GDS was 2.5 (±2.5). 14.0% of the individuals were socially engaged at least once a week. After 4.5 years (FU wave 5), the proportion of unmarried individuals significantly increased as many spouses died. Other sociodemographic variables remained nearly constant. Additionally, while functional impairment increased, the mean comorbidity score decreased.

**Table 1:** Descriptive Statistics over Time (Follow-Up Waves 2–5)

Variables	Follow-Up Wave 2 (*n* = 2364)	Follow-Up Wave 3 (*n* = 1827)	Follow-Up Wave 4 (*n* = 1453)	Follow-Up Wave 5 (*n* = 1174)
Age: Mean (SD)	82.5 (3.4)	83.9 (3.3)	85.2 (3.2)	86.7 (3.0)
Female: N (%)	1556 (65.8)	1203 (65.9)	963 (66.3)	790 (67.3)
Education: N (%)				
Low Education	1431 (60.5)	1092 (59.8)	838 (57.7)	665 (56.6)
Middle Education	656 (27.8)	517 (28.3)	438 (30.1)	356 (30.3)
High Education	277 (11.7)	218 (11.9)	177 (12.2)	153 (13.0)
Unmarried: N (%)	1449 (61.4)	1193 (65.4)	966 (66.5)	832 (70.9)
Living alone in private household: N (%)	1216 (51.4)	959 (52.5)	773 (53.2)	606 (51.6)
Comorbidity (Weighted count score): Mean (SD)	4.5 (3.8)	4.4 (3.7)	4.5 (3.8)	3.9 (3.7)
Functional status (IADL): Mean (SD)	6.8 (1.7)	6.8 (1.7)	6.6 (1.8)	6.3 (2.0)
HRQoL (EQ-VAS): Mean (SD)	65.5 (17.9)	64.8 (17.5)	64.3 (17.5)	62.9 (18.9)
Depressive symptoms (Geriatric Depression Scale): Mean (SD)	2.5 (2.5)	2.4 (2.3)	2.5 (2.4)	2.7 (2.5)
Social engagement: N (%)				
No social engagement	2031 (86.0)	1662 (91.4)	1318 (91.2)	1090 (93.6)
Once a week	175 (7.4)	85 (4.7)	69 (4.8)	41 (3.5)
Several times a week	116 (4.9)	54 (3.0)	40 (2.8)	25 (2.1)
Daily social engagement	39 (1.7)	16 (0.9)	17 (1.2)	8 (0.7)

Furthermore, it is worth mentioning that between FU wave 2 and FU wave 5 165 individuals started social engagement in this longitudinal study (i.e. intraindividual changes from ‘no social engagement’ to ‘social engagement’ (once a week, several times a week or daily)). Contrarily, for example, 112 individuals changed from social engagement several times a week to no social engagement) (further details to intraindividual changes are available upon request).

### Regression analysis

Adjusting for age, marital status, functional status (IADL) and chronic diseases, FE regressions revealed that the onset of social engagement several times a week (Ref.: no social engagement) markedly increased HRQoL in the total sample (β = 4.1, *p* < .001) and in women (β = 6.2, *p* < .001) (see Table 2). In other words: The loss of social engagement substantially reduced HRQoL in the total sample and in women. It is worth mentioning that changes in social engagement did not affect HRQoL in men (with significant interaction term: gender x onset of social engagement several times a week, *p* < .05).

**Table 2:** Predictors of HRQoL (EQ-VAS): Results of fixed effects regression (Follow-up waves 2–5)

Independent variables	(1)	(2)	(3)	(4)
	HRQOL – Total sample	HRQOL - Women	HRQOL - Men	HRQOL - Interaction
Increasing age	−1.049***	−1.057***	−1.082***	−1.061***
	(0.106)	(0.144)	(0.172)	(0.106)
Loss of spouse	1.882	0.875	3.591*	1.961
	(1.195)	(1.606)	(1.752)	(1.195)
Onset of social engagement once a week (Ref.: No social engagement)	0.188	−0.244	0.989	−0.232
	(0.954)	(1.217)	(1.501)	(1.216)
Onset of social engagement several times a week	4.056***	6.150***	0.235	6.165***
	(1.220)	(1.483)	(2.031)	(1.480)
Onset of daily social engagement	1.095	−0.919	4.150	−0.853
	(1.864)	(2.312)	(3.071)	(2.305)
Interaction: Gender x onset of social engagement once a week				1.186
				(1.924)
Gender x onset of social engagement several times a week				−5.929*
				(2.493)
Interaction: Gender x onset of daily social engagement				4.775
				(3.793)
Decreasing functional impairment (IADL)	0.591**	0.556*	0.606*	0.581**
	(0.186)	(0.274)	(0.260)	(0.186)
Comorbidity (Weighted score)	−0.121	−0.120	−0.113	−0.116
	(0.0964)	(0.129)	(0.145)	(0.0965)
Constant	148.0***	148.3***	153.1***	149.0***
	(9.104)	(12.95)	(14.21)	(9.088)
Observations	6816	4511	2305	6816
R^2^	0.036	0.039	0.035	0.038
Number of Individuals	2381	1565	816	2381

Comments: Beta-Coefficients were reported; Cluster-robust standard errors in parentheses. ***p < 0.001, **p < 0.01, *p < 0.05, +*p* < 0.10. Observations with missing values were dropped (listwise deletion)

Moreover, FE regressions revealed that the onset of social engagement decreased depressive sympoms in the total sample (onset of social engagement once a week: β = −.3, *p* < .01; onset of social engagement several times a week: β = −.3, *p* < .01) and in women (onset of social engagement once a week: β = −.4, *p* < .01; onset of social engagement several times a week: β = −.3, *p* < .05) (see Table 3). Even though changes in social engagement did not affect depressive symptoms in men, the interaction terms (gender x social engagement) did not achieve statistical significance.

**Table 3:** Predictors of depressive symptoms (GDS): Results of fixed effects regression (Follow-up waves 2–5)

Independent variables	(1)	(2)	(3)	(4)
	Depressive symptoms – Total sample	Depressive symptoms - Women	Depressive symptoms - Men	Depressive symptoms - Interaction
Increasing age	0.0711***	0.0416*	0.0978***	0.0710***
	(0.0118)	(0.0164)	(0.0184)	(0.0119)
Loss of spouse	0.101	0.128	0.0557	0.103
	(0.164)	(0.219)	(0.243)	(0.164)
Onset of social engagement once a week (Ref.: No social engagement)	−0.346**	−0.389**	−0.250+	−0.381**
	(0.106)	(0.144)	(0.132)	(0.145)
Onset of social engagement several times a week	−0.274**	−0.307*	−0.175	−0.307*
	(0.106)	(0.128)	(0.186)	(0.127)
Onset of daily social engagement	−0.219	−0.329	−0.100	−0.310
	(0.209)	(0.305)	(0.266)	(0.302)
Interaction: Gender x onset of social engagement once a week				0.109
				(0.198)
Gender x onset of social engagement several times a week				0.0921
				(0.224)
Interaction: Gender x onset of daily social engagement				0.225
				(0.407)
Decreasing functional impairment (IADL)	−0.155***	−0.205***	−0.0916**	−0.155***
	(0.0238)	(0.0350)	(0.0287)	(0.0239)
Comorbidity (Weighted score)	0.0310**	0.0345*	0.0215	0.0310**
	(0.0102)	(0.0139)	(0.0141)	(0.0102)
Constant	−2.600*	0.399	−5.519***	−2.601*
	(1.008)	(1.471)	(1.516)	(1.013)
Observations	7010	4658	2352	7010
R^2^	0.046	0.054	0.035	0.046
Number of Individuals	2391	1572	819	2391

Comments: Beta-Coefficients were reported; Cluster-robust standard errors in parentheses. *** *p* < 0.001, ** *p* < 0.01, * *p* < 0.05, + *p* < 0.10. Observations with missing values were dropped (listwise deletion)

## Discussion

This study investigated the impact of social engagement (engagement in the church, as a volunteer, in a party, or in a club) on HRQoL and depressive symptoms in late life in Germany. After adjusting for main control variables such as age, functional status and chronic diseases, longitudinal regressions showed that the onset of social engagement markedly increased HRQoL as well as considerably decreased depressive symptoms in the total sample and in women, but not men.

Several cross-sectional studies showed that social engagement is positively associated with HRQoL and less depressive symptoms in late life. Moreover, longitudinal studies found significant associations between social engagement and HRQoL as well as depressive symptoms in old age. For instance, using the Singapore longitudinal ageing studies, Schwingel [13] showed that volunteering retirees have less depressive symptoms compared with non-volunteering retirees at baseline and 2-year follow-up. Additionally, Menec [12] investigated the association between respective activities at baseline and subsequent outcomes (well-being, function and mortality). By using logistic regressions, Menec [12] found that volunteer work at baseline (1990) was predictive of better function 6 years later. Data were derived from the Aging in Manitoba (AIM) Study which is a long-running representative sample of older adults living in Manitoba (Canada). Furthermore, by using latent class growth curve models, Hong et al. [16] showed that volunteering was associated with changes in depressive symptoms in old age. They used three waves (1994–2000) from the Longitudinal Study on Aging (LSOA) II which is a nationally representative sample of community-dwelling individuals aged 70 years and above. However, they used a single item (how often they felt sad or depressed in the past 12 months using a 4-point scale) to quantify depression. Furthermore, by using the generalized estimating equations method, Morrow-Howell et al. [17] found that older adults who volunteer report higher levels of self-rated health (1–5, excellent to poor) and less depressive symptoms (modified Center for Epidemiological Studies Depression Scale (CES-D)). They used three waves (1986, 1989, 1994) from the Americans’ Changing Lives (ACL) Study. The ACL Study is a panel survey (in wave 1, 1669 older adults were included in the analysis) with an oversampling of people aged 60 and above.

Our study adds to the growing body of evidence that social engagement in late life affects HRQoL and depressive symptoms. By focusing solely on intraindividual changes - which is very important in HRQoL and depressive symptoms research - our findings extend previous studies. Thus, insights into the causal relationship can be derived. Our key findings - social engagement affects HRQoL and depressive symptoms - might be explained by a sense of competence and feelings of usefulness that are accompanied by social engagement. These variables are related to mastery and a sense of control: Factors that are in turn associated with depressive symptoms and HRQoL.

Like other studies [17], data suggest a nonlinear relationship between frequency of volunteering and our health outcomes, indicating that there might be an optimal frequency of social engagement in late life. From a public health perspective, it is worth emphasizing that the onset of *weekly* social engagement already decreased depressive symptoms. Thus, even *modest amounts* of social engagement might be beneficial. This information can be used to let voluntary activities sound more attractive. However, more research is required on this issue as we did not know the exact hours per week socially engaged.

The non-significant impact of social engagement on HRQoL and depressive symptoms among men might be explained by the types of activities examined in the present study. More specifically, activities that are more informal might be beneficial to men. However, this should be clarified in future studies.

This is the first longitudinal study examining the impact of social engagement on HRQoL and depressive symptoms in old age in Germany. By using FE regressions, (1) time-constant heterogeneity can be taken into account, providing unbiased estimates (under the assumption of strict exogeneity). Moreover, (2) insights into the causal relationship can be derived. Furthermore, because individuals were recruited via GP offices and almost every individual in old age has regularly GP visits in Germany, the sample can be considered as nearly representative. Additionally, HRQoL was quantified by using the validated EQ-VAS. Moreover, depressive symptoms was assessed by the well-validated GDS.

However, due to data restrictions, we were not able to distinguish between the different areas of social engagement (e.g. engagement in the church, as a volunteer, in a party, or in a club). There might be some differences in the effect on our outcome variables. Moreover, our estimates might be biased downwards for reasons of panel attrition in the AgeCoDe study.

## Conclusion

Our data underline the importance of social engagement in late life. Thus, encouraging individuals to start, maintain and expand social engagement might help to maintain and improve HRQoL as well as to decrease depressive symptoms in old age. Moreover, social engagement in late life may also be beneficial for society as a whole [17]. Future research should disentangle which aspects of social engagement in late life are associated with HRQoL and depressive symptoms to understand more fully the conditions that influence the effect of social engagement on HRQoL and depressive symptoms in late life [17].

## Abbreviations

ACL: Americans’ Changing Lives
AgeCoDe: German Study on Ageing, Cognition and Dementia in Primary Care Patients
AIM: Aging in Manitoba
CASMIN: Comparative Analysis of Social Mobility in Industrial Nations
CES-D: Center for Epidemiological Studies Depression Scale
FE: Fixed effects
GDS: Geriatric Depression Scale
GP: General Practitioner
HRQoL: Health-related quality of life
IADL: Instrumental Activities of Daily Living
LSOA: Longitudinal Study on Aging
NH: Nursing home
RE: Random effects
VAS: Visual Analogue Scale
